# Pharmacokinetic Comparison of Seven Major Bio-Active Components in Normal and Blood Stasis Rats after Oral Administration of Herb Pair Danggui-Honghua by UPLC-TQ/MS

**DOI:** 10.3390/molecules22101746

**Published:** 2017-10-17

**Authors:** Yi Jin, Yu-Ping Tang, Zhen-Hua Zhu, Er-Xin Shang, Han-Qing Pang, Xu-Qin Shi, Yan-Yan Chen, Jin Wang, Xing Chang, An Kang, Gui-Sheng Zhou, Ya-Jun Shi, Jin Sun, Zhi-Shu Tang, Shao-Ping Li, Jin-Ao Duan

**Affiliations:** 1 Shaanxi Collaborative Innovation Center of Chinese Medicinal Resources Industrialization and College of Pharmacy, Shaanxi University of Chinese Medicine, Xianyang 712046, China; jinyixihuan@163.com (Y.J.); chenyanyan---c@163.com (Y.-Y.C.); wangjing19890126@126.com (J.W.); changxing_2011@163.com (X.C.); iottsyj@163.com (Y.-J.S.); ph.175@163.com (J.S.); tzs6565@163.com (Z.-S.T.); 2 Jiangsu Collaborative Innovation Center of Chinese Medicinal Resources Industrialization, and Jiangsu Key Laboratory for High Technology Research of TCM Formulae, and National and Local Collaborative Engineering Center of Chinese Medicinal Resources Industrialization and Formulae Innovative Medicine, Nanjing University of Chinese Medicine, Nanjing 210023, China; 18913133908@163.com (Z.-H.Z.); shex@njucm.edu.cn (E.-X.S.); hanqingpang@126.com (H.Q.P.); shixuqin@126.com (X.-Q.S.); zhouguisheng1@126.com (G.-S.Z.); dja@njucm.edu.cn (J.-A.D.); 3 State Key Laboratory of Quality Research in Chinese Medicine, Institute of Chinese Medical Sciences, University of Macau, Macau 999078, China; spli@umac.mo

**Keywords:** Danggui, *Angelica sinensis*, honghua, *Carthamustinctorius*, herb pair, pharmacokinetics

## Abstract

The compatibility between Danggui (Angelicae Sinensis Radix) and Honghua (Carthami Flos) is a known herb pair, which could activate blood circulation and dissipate blood stasis effects. In this paper, we quantified seven main bio-active components (hydroxysafflor yellow A, caffeic acid, *p*-coumaric acid, kaempferol-3-*O*-rutinoside, ferulic acid, 3-*n*-butylphthalide, and ligustilide) in plasma samples in vivo by UPLC-TQ/MS method and investigatedwhether the pharmacokinetic (PK) behaviors of the seven components could be altered in blood stasis rats after oral administration of the Gui-Hong extracts. It was found that the *C*_max_ and *AUC*_0-t_ of these components in blood stasis rats had increasing tendency compared with normal rats. Most components in model and normal rats had significant difference in some pharmacokinetic parameters, which indicated that the metabolism enzymes and transporters involved in the metabolism and disposition of these bio-active componentsmay bealtered in blood stasis rats. This study was the first report about the pharmacokinetic investigation between normal and blood stasis rats after oral administrationof Gui-Hong extracts, and these results are important and valuable for better clinical applications of Gui-Hong herb pair and relatedTCM formulae.

## 1. Introduction

Danggui (Angelicae Sinensis Radix) is the dried radix of *Angelica sinensis* (Oliv.) Diels (Umbelliferae) and Honghua (CarthamiFlos) is the dried flower of *Carthamus* *tinctorius* L. (Compositae). The compatibility between Danggui and Honghua could be found in Danggui-Honghua Decoction of *Shanghandabai*. It is an ancient and classic formula comprised of Danggui and Honghua, and it is also a famous herb pair (Gui-Hong), which has the activating blood circulation and dissipating blood stasis effects [1].

To date, pharmacological studies and chemical investigation have found that the aromatic acids, phthalides and quinochalcone *C*-glycosides were the main constitutes responsible for the herb pair’bio-activities [2,3]. Pharmacokinetic studieson these components havebeen partly demonstrated [4,5,6]. However, there arefew reports about the pharmacokinetic characteristics ofthe herb pair. The pharmacokinetic process of herbs will be affectedby the co-usedherbs when used as an herb pair or herb formulae [7].

Blood stasis syndrome isan important cause of many diseases, including myocardial ischemia anddysmenorrheal.There is no doubt that these pathological alternationsmay significantlyinfluence theprotein/mRNA expressions or activities of metabolic enzymes and transportersinvolved in the absorption or disposition of these active components in the Gui-Hong herb pair [8]. Considering the popular use of the Gui-Hong herb pair in the treatment of blood stasis syndrome, in this study, a sensitive and reliable ultra-high-performance liquid chromatography coupled with a triple quadrupole electrospray tandem mass spectrometry (UPLC-MS/MS) method which can be used tosimultaneously quantify seven main bio-active components (hydroxysafflor yellow A, caffeic acid, *p*-coumaric acid, kaempferol-3-*O*-rutinoside, ferulic acid, 3-*n*-butylphthalide, and ligustilide) in plasma samples was developed. Additionally, the pharmacokinetic (PK) parameters of the seven components in normal and blood stasis rats after oral administration of Gui-Hong extracts were compared.

## 2. Results and Discussion

### 2.1. The Component Contents in Gui-Hong Extracts

A validated UPLC-TQ/MS method was employed to quantifythe contents of the main constitutes in Gui-Hong extracts. The results showed that Gui-Hong extracts (0.405 g/mL) contained 144.075 μg/mL of HSYA, 3.758 μg/mL of caffeic acid, 4.350 μg/mL of *p*-coumaric acid, 33.844 μg/mL of kaempferol-3-*O*-rutinoside, 31.647 μg/mL of ferulic acid, 1.583 μg/mL of 3-*n*-butylphthalide, and 3.175 μg/mL of ligustilide.

### 2.2. The Observation on Haemorheology of Rats

The haemorheological indices of the rats in these two groups are shown in Table 1, including whole blood viscosity (WBV), plasma viscosity (PV), erythrocyte sedimentation rate (ESR), andhematocrit (HCT). These indiceswere the main diagnostic criteria of blood stasis syndrome. Results showed that WBV (200 s^−1^, 30 s^−1^, 5 s^−1^), PV, ESR, and HCT in blood stasis rats increased significantly (*p*< 0.05, *p*< 0.01) when compared with the normal rats, which indicatedthat the blood stasis animal model was successfully established.

### 2.3. Method Validation

#### 2.3.1. Specificity

**Figure 1:**
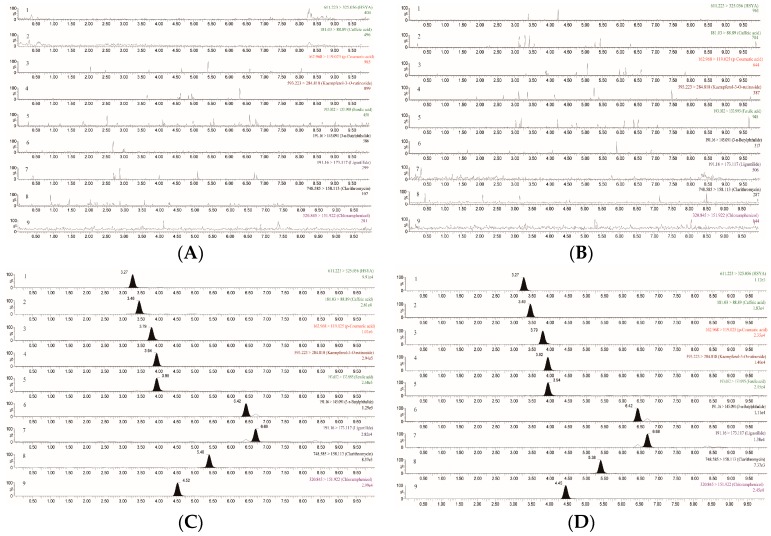
Typical MRM chromatograms of the seven components in rats: (**A**) blank plasma sample from a normal rat; (**B**) blank plasma sample from a model rat; (**C**) blank plasma samples spiked with standard mixtures and internal standards; and (**D**) ratplasma samples collected after oral administration of Gui-Hong extracts. Note: hydroxysafflor yellow A (**1**), caffeic acid (**2**), *p*-coumaric acid (**3**), kaempferol-3-*O*-rutinoside (**4**), ferulic acid (**5**), 3-*n*-butylphthalide (**6**), ligustilide (**7**), chloramphenicol (**8**), and clarithromycin (**9**).

No interference could beobserved at the elutingtimes of either analytes or IS in blankplasma samples from normal or blood stasis rats, which indicated thatthe method exhibited good specificity. Typical MRM chromatograms were shown in Figure 1.

#### 2.3.2. Linearity and LLOQ

Linear equation ofthese seven calibration curves, linear ranges and LLOQs of these seven analytes using blank plasma samples from normal rats arepresented in Table 2.

#### 2.3.3. Precision and Accuracy

The developed method showed an acceptable precision and accuracy inthe intra-day and inter-day test. All of these results were expectedto meet the required variable limits and the detailed data areshown in Table 3.

#### 2.3.4. Extraction Recovery and Matrix Effect

Extraction recovery and matrix effect of the seven components could be seen in Table 3. Average extraction recovery of the two ISs, chloramphenicol and clarithromycin, were 86.11 ± 1.20% and 85.85 ± 2.74%, respectively.The matrix effect of these ISs were 87.67 ± 1.01% and 86.15 ± 1.81%.

#### 2.3.5. Stability

The stability of all these seven analytes in rat plasma under different storage conditions was evaluated. As shown in Table 3, the results indicatedthat these analytes were all stable in these different conditions.

### 2.4. Pharmacokinetic Study

#### 2.4.1. Comparison of Pharmacokinetic Parameters of Each Gui-Hong Component in Normal and Blood Stasis Rats

Blood stasis syndrome is a kind of pathological state in blood circulation and causes the development of many diseases. Based on TCM theory, it is characterized with a slowing or pooling blood mainly owingto the disruption of heart Qi, and now it is often understood to be the hematological disorders (e.g., a rise in whole blood and plasma viscosity) [9,10]. Thus, we investigate the pharmacokinetic behaviors of the main bioactive constitutes in Gui-Hong herb pair in blood stasis rats induced by subcutaneously injecting with adrenaline hydrochloride.

**Figure 2:**
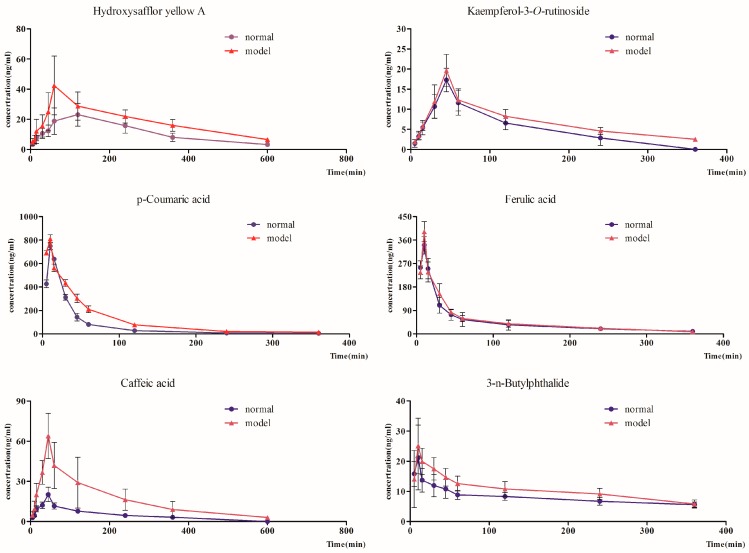

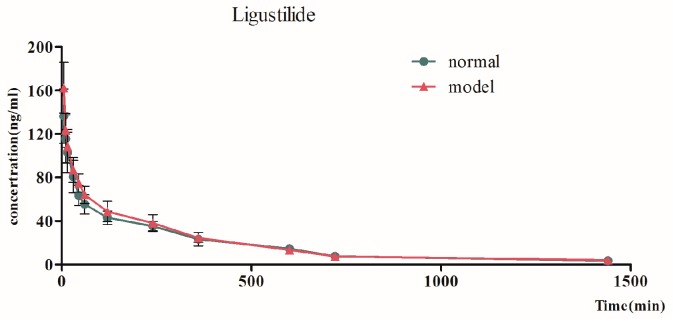
Mean plasma concentration-time curves of seven components after oral administration of Gui-Hong extracts to normal and blood stasis rats.

The mean plasma concentration-time profiles of the seven components were illustrated in Figure 2 and the main pharmacokinetic parameters were calculated and summarized in Table 4, respectively.

Pharmacokinetic parameters of HSYA of blood stasis rats exhibited higher *C*_max_, *T*_1/2z_, *AUC*_0-t_, *MRT*_0-t_, and *AUC*_0-∞_, and lower *T*_max_. Among these data, *T*_max_, *AUC*_0-t_, *AUC*_0-∞_, and *T*_1/2z_ had significant difference. As previously work indicated that HSYA was mainly absorbed through the small intestine, while poor blood circulation might prolong the retention time of HSYA in the small intestine, thus eventually led to an increased bioavailability of HSYA [5]. Additionally, slowed blood circulation may decrease the liver perfusion which may lead to decreased hydroxylation, methylation, acetylation, and glucuronidation of the HSYA in liver. This decreased drug metabolism may significantly increase the bioavailability of HSYA in blood stasis rats [11].

For caffeic acid, the plasma samples from the blood stasis rats showed higher *C*_max_, *AUC*_0-t_, *MRT*_0-t_, *T*_1/2z_, and *AUC*_0-∞_. *T*_max_ of caffeic acid in normal and blood stasis rats was same. Meanwhile, the *C*_max_, *AUC*_0-t_, *MRT*_0-t_, and *AUC*_0-∞_ of caffeic acid had significant difference. Catechol-*O*-methyltransferase (COMT) mediated *O*-methylation plays an important role in the metabolism of caffeic acid in rat hepatocytes. Blood stasis syndromesinduced by adrenaline hydrochloride or occlusion of the left anterior descending coronary arteryhave shown decreased COMT activity in liver plasma and the heart [12,13]. The increased bioavailability of caffeic acid may partially contribute to the decreased liver and blood metabolism [14,15].

For *p*-coumaric acid, the model samples revealed higher *C*_max_, *AUC*_0-t_, *MRT*_0-t_, *T*_1/2z_, and *AUC*_0-∞_. And the *C*_max_, *AUC*_0-t_, *MRT*_0-t_, and *AUC*_0-∞_ of *p*-coumaric acid had a significant difference. Our previously work has demonstrated that the pharmacokinetic behavior of *p*-coumaric acid may be altered after compatibility in rats [16]. While in this study, the result indicated that the absorption of *p*-coumaric acid could be increased and the process of elimination was slowed in thepathological state.

As for kaempferol-3-*O*-rutinoside, the model samples showed higher *C*_max_, *T*_1/2z_, *AUC*_0-t_, *MRT*_0-t_, and *AUC*_0-∞_ compared with normal rats, and the *T*_1/2z_, *AUC*_0-t_, *MRT*_0-t_, and *AUC*_0-∞_ of kaempferol-3-*O*-rutinoside had significant difference. Kaempferol-3-*O*-rutinoside is a kind of flavonoid glycosides. A series of reasons such as high polarity, large molecules, etc., will result in the low bioavailability of kaempferol-3-*O*-rutinosideafter oral administration [17].

For ferulic acid with similar chemical structure of *p*-coumaric acid, the model rats implied higher *C*_max_, *AUC*_0-t_, and *AUC*_0-∞_, and lower *T*_1/2z_ and *MRT*_0-t_. The *C*_max_ of ferulic acid had a significant difference. Additionally, *T*_max_ of ferulic acid in these two groups was the same, which implied that ferulic acid absorbed more rapidly in blood stasis rats.

When looking at 3-*n*-butylphthalide, the model rats showed lower *T*_1/2z_ and *MRT*_0-t_, and higher *C*_max_, *AUC*_0-t_, and *AUC*_0-∞_. Moreover, the *T*_1/2z_ and *AUC*_0-t_ of 3-*n*-butylphthalide had a significant difference. Since 3-*n*-butylphthalide was mainly metabolized by CYP2E1, 2C11 and 3A1/2 in rats [18], which referred that model rats could change the eliminated time of 3-*n*-butylphthalide may due to the changes in the enzymes. Also, Gui-Hong herb pair also could up-regulate the CYP1A and CYP3A mRNA expression, but decrease the CYP2E1 mRNA expression [19]. Thus, further work need to be conducted to find whether the mRNA or protein expression of these metabolism enzymes were changed in this pathological states, especially when fed with the Gui-Hong herb pair.

Finally, for ligustilide, the model rats exhibited higher *C*_max_, *AUC*_0-t_, and *AUC*_0-∞_, and lower *MRT*_0-t_ and *T*_1/2z_. The result implied that blood stasis model rats could speed up the time and extent of absorption of ligustilide, however, there was no significant difference between normal and model rats.Further studies may be helpful in providing additional insight.

In this study, we mainly focused on the changed pharmacokinetic behavior of these seven active components in the herb pair in blood stasis model rats. In order to uncover the underlying mechanisms, the activity, mRNA and protein expression of these enzymes and transporters need to further studied in our ongoing works.

#### 2.4.2. The Pharmacokinetic Comparison of Different Types of Gui-Hong Components in Normal and Blood Stasis Rats

For further study, we divided the seven major bio-active components into three different types. The total *T*_max_ and *AUC*_0-t_ calculated from the three different types of components in Gui-Hong extracts as two integrated PK parameters were used in order to compare their pharmacokinetic profiles of normal and blood stasis rats. The total *T*_max_ and *AUC*_0-t_ of these aromatic acids, including caffeic acid, ferulic acid, and *p*-coumaric acid, in normal rats were 2.583 h, 147.547 ng mL^−1^ h while that in model rats were 1.917 h, 228.747 ng mL^−1^ h, respectively. The total *T*_max_ and *AUC*_0-t_ of flavonoid glycosides (HSYA, kaempferol-3-*O*-rutinoside) were 1.084 h, 744.527 ng mL^−1^ h in normal rats and 1.084 h, 1169.482 ng mL^−1^ h in model rats, respectively. The total *T*_max_ and *AUC*_0-t_ ofphthalides (3-*n*-butylphthalide, ligustilide) were 0.25 h, 480.579 ng mL^−1^ h in normal rats and 0.25 h, 524.676 ng mL^−1^ h in model rats, respectively. It was found that only the *T*_max_ of aromatic acids in model rats was shorter than that in normal rats. The *AUC*_0-t_ of the three different types of components in the model rats were all larger than in normal rats, which suggested that these components had higher exposure levels in model rats than in normal rats.

## 3. Experimental

### 3.1. Chemicals and Regents

The radix of *Angelica sinensis* (Oliv.) Diels (Umbelliferae) was collected at Min County, Gansu Province, China, in July 2014. The flos of *Carthamus* *tinctorius* L. (Compositae) was collected at Yili County, Xinjiang Province, China, in March 2014. All of these herb medicineswere identified by Dr. Hui Yan in our group.The voucher specimens (Nos. NJUTCM-20111009 and NJUTCM-20111018) were deposited in the Herbarium of Nanjing University of Chinese Medicine.

**Figure 3:**
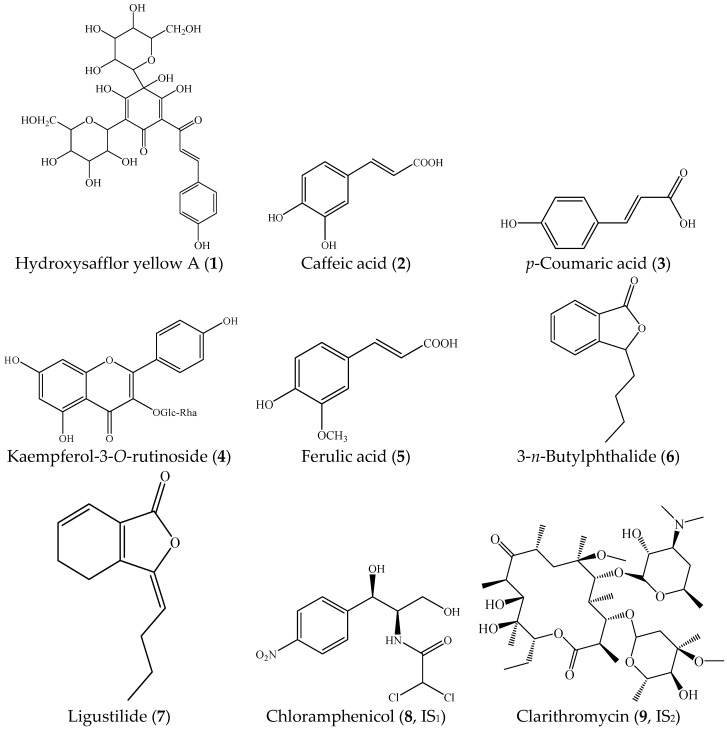
Chemical structures of hydroxysafflor yellow A (**1**), caffeic acid (**2**), *p*-coumaric acid (**3**), kaempferol-3-*O*-rutinoside (**4**), ferulic acid (**5**), 3-*n*-butylphthalide (**6**), ligustilide (**7**), chloramphenicol (**8**), and clarithromycin (**9**).

Reference standards of hydroxysafflor yellow A (HSYA) (1, 98%), caffeic acid (2, 98%), *p*-coumaric acid (3, 98%), kaempferol-3-*O*-rutinoside (4, 98%), ferulic acid (5, 98%), 3-*n*-butylphthalide (6, 98%), and ligustilide (7, 98%) were supplied by Chengdu Chroma-Biotechnology Co., Ltd. (Chengdu, China). Chloramphenicol (8, IS_1_) and clarithromycin (9, IS_2_) were purchased from the National Institute for the Control of Pharmaceutical and Biological products (Beijing, China). Their chemical structures arepresented in Figure 3. Acetonitrile, methanol, and acetic acid (HPLC grade) were purchased from Merck KGaA (Darmstadt, Germany). Ultra-pure water was purified by an EPED super purification system (Nanjing, China). Adrenaline hydrochloride was purchased from Harvest pharmaceutical Co., Ltd., Shanghai, China (batch number: W150102, Shanghai, China).

### 3.2. Extract Preparation

A total 500 g Gui-Hong (1:1, *w*/*w*) cut into pieces were extracted with boiling water (1:8, *w*/*v*) for once with a total time of 2 h, and filtered through gauze. Then the residue was refluxed with boiling water (1:6, *w*/*v*) twice, with 1.5 h for each time. Allthree filtrates were merged and evaporated with rotary evaporation under vacuum at 50 °C, and then the Gui-Hong extract could be obtained.

### 3.3. Pharmacokinetic Study

Female Sprague-Dawley (SD) rats (200 ± 20 g) were purchasedfrom Shanghai SLAC Laboratory Animal Co. Ltd. (Shanghai, China). The rats were kept in a controlledenvironmental condition (temperature: 20 ± 2 °C, humidity: 60 ± 5%) for oneweek untilthe experiments started. Animal welfare and experimental procedures were strictly in accordance with the *Guide for the Care and Use of Laboratory Animals* (US National Research Council, 1996) and the related ethics regulations of Nanjing University of Chinese Medicine.

Rats were randomly divided into two groups (*n* = 6): a normal control group administrated with Gui-Hong extracts, and an acute blood stasis model group also orally administered with Gui-Hong extracts. The acute blood stasis rats were induced by injectingwith adrenaline hydrochloride (0.8 mg/kg). 2 hlater, the rats were soaked in ice-water for 4 min with their headskeeping above the surface. After 2 h again, the rats were then injected with adrenaline hydrochloride again [9]. All rats were fasted for 12 h with free access to water prior to the experiments. Both normal and blood stasis rats were orally administered Gui-Hong extracts at a dosage of 4.05 g/kg (4.05 g crude herbs per 1 kg rat body weight) which was homogeneously dissolved and dispersed in ultrapure water. The animal dose of the Gui-Hong extracts was extrapolated from the human daily dose, using the body surface area normalization method. The formula for dose translation was as follows: human dose of crude herbs in clinic × 0.018/200 × 1000 × the multiple of clinical equivalency dose [9]. The dose of Gui-Hong extracts was equivalent to three times the adult daily dose of the Gui-Hong herb pair (15 g, from Tao-Hong-Si-Wu-Tang in which Danggui and Honghua were 9 g and 6 g, respectively) crude herbs based on the TCM prescription. Then 0.3 mL blood samples were collected into the centrifuge tubes which contained 0.03 mL EDTA-2Na from each rat at different time points (5, 10, 15, 30, 45, 60, 120, 240, 360, 600, 720, and 1440 min). Blood samples were centrifuged at 3000 rpm for 10 min at 4 °C and the supernatant was transferred and stored at −70 °C until analysis.

### 3.4. Chromatography and Mass Spectrometry Conditions

Chromatographic separation was carried out on a Thermo Hypersil Gold C_18_ column (2.1 mm × 50 mm, 1.9 μm) maintained at 35 °C. The mobile phase gradient conditions consisted of 0.1% aqueous acetic acid (A) and acetonitrile (B): 5% B (1.0 min)-40%B (5.0 min)-95% B (8.0 min)-95% B (9.0 min)-5% B (9.2 min)-5% B (10.0 min). The flow rate was set at 0.4 mL/min. The auto-sampler was conditioned at 4 °C and the sample injection volume was 2 μL. The ESI source was set in both positive and negative ionization mode. The scanning mode was set multiple reaction monitoring (MRM) mode and the retention time (*t*_R_), optimized MS/MS transitions, cone voltage and collision energy of each component were shown in Table 5. The TQ mass spectrometer was operated with a capillary voltage of 3 kV, a source temperature of 150 °C, and a desolvation temperature of 500 °C. MassLynx and DAS 2.0 (Waters, Corp., Milford, MA, USA) software were employed to analyze the data.

### 3.5. Preparation of Calibration Standards and Quality Control (QC) Samples

The stock solutions of IS_1_ (189 μg/mL), IS_2_ (181 μg/mL), HSYA (225 μg/mL), caffeic acid (229 μg/mL), *p*-coumaric acid (213 μg/mL), kaempferol-3-*O*-rutinoside (137 μg/mL), ferulic acid (180 μg/mL), 3-*n*-butylphthalide (246 μg/mL), and ligustilide (386 μg/mL) were prepared in methanol, respectively. Then the series of working solutionswere obtained by further dilutionwith methanol.

Low, middle, and high concentrations of quality control (QC) samples were independently prepared at concentrations of 2.25, 22.5, and 180 ng/mL for HSYA; 2.29, 22.9, and 183.2 ng/mL for caffeic acid; 2.13, 106.5, and 852 ng/mL for p-coumaric acid; 2.74, 68.5, and 548 ng/mL for kaempferol-3-*O*-rutinoside; 9, 90, and 720 ng/mL for ferulic acid; 2.46, 24.6, and 196.8 ng/mL for 3-*n*-butylphthalide; and 1.93, 38.6, and 308.8 ng/mL for ligustilide using the same method as for the calibration samples. All stock solutions and working solutions were stored at −20 °C in dark until use.

### 3.6. Plasma Sample Preparation

Plasma samples were thawed at 37°Cbefore analysis. Then the 60 μL mixture of IS_1_ and IS_2_ solution (4.725 μg/mL of IS_1_ and 90.5 ng/mL of IS_2_) and 540 μL of methanol were added to the plasma sample (200 μL) in a 1.5 mL Eppendorf tube. The mixture was vortexed for 5 min and centrifuged at 13,000 rpm for 10 min at 4 °C to deproteinize. The supernatant was then transferred into a new 1.5 mL centrifuge tube and evaporated to dryness under vacuum with a Labconco CentriVap concentrator (Kansas City, MO, USA). The residue was reconstituted in 200 μL 80% methanol solution, and the mixtures were vortexed for 5 min, and centrifuged at 13,000 rpm for 10 min at 4 °C. Finally, 2 μL of the supernatant was injected into the UPLC-TQ/MS system for analysis.

### 3.7. Method Validation

#### 3.7.1. Specificity

The specificity was calculated by comparing blank plasma samples from normal rats and blood stasis rats, blank plasma samples spiked with standards and internal standards, and plasma samplescollected after oral administration of Gui-Hong extracts and spiked with ISs.

#### 3.7.2. Linearity and Lower Limit of Quantification (LLOQ)

For the calibration curve, the mixture stock solution was diluted with methanol to make a series of working solutions. The calibration samples were prepared independently by adding a series of different concentration working solutions (200 μL), IS solution (60 μL), and 340 μL methanol to blank rat plasma (200 μL) to determine linearity and the lower limit of quantification (LLOQ).

#### 3.7.3. Precision and Accuracy

The precision and accuracy of the method wereevaluated by analysisof three QC samples. The precision was determined from inter-day and intra-day using low, middle, and high concentrations. The precision was expressed by relative standard deviation (RSD%), and the accuracy by relative error (RE%).

#### 3.7.4. Recovery and Matrix Effects

The extraction recoveries of analytes were determined by comparing the peak areas of the QC samples pre-spiked in blank plasma with those post-spiked in blank plasma (*n* = 6). The matrix effect was determined by comparing the peak areas of the QC samples pre-spiked in blank plasma with those in the mobile phase (*n* = 6).

#### 3.7.5. Stability

The stability of analytes in the plasma was performed byusing the QC samples under three conditions: (1) short-term stability, QC samples (*n* = 6) werestored at room temperature for 12 h and at refrigerated (4 °C) for 24 h; (2) long-term stability, QC samples (*n* = 6) were stored at −80 °C for 20 days; (3) three freeze–thaw cycles stability, QC samples (*n* = 6) were detected after three cycles of freezing (−20 °C) and thawing (ambient temperature).

## 4. Conclusions

In this study, the developed UPLC–MS/MS method for the quantification of HSYA, caffeic acid, *p*-coumaric acid, kaempferol-3-*O*-rutinoside, ferulic acid, 3-*n*-butylphthalide, and ligustilide in normal and model rat plasma is suitable for pharmacokinetic study. Pharmacokinetic results indicated that the seven bio-active components had obvious differences in some pharmacokinetic characteristics, suggesting that the drug metabolism enzymes might be altered in animals with blood stasis syndrome. The changed pharmacokinetic processes of the herb pair in normal and pathological conditions could help us explain and predict various events related to the efficacy and adverse reaction of Gui-Hong extract, and for better clinical applications of the Gui-Hong herb pair and relatedTCM formulae.

## Figures and Tables

**Table 1:** The haemorheological indices of normal and blood stasis rats.

Group	WBV/mPa·s				PV/mPa·s (200 s^−1^)	ESR/mm·h^−1^	HCT/L·L^−1^
	200 s^−1^	30 s^−1^	5 s^−1^	1 s^−1^			
Control	3.22 ± 0.16	3.78 ± 0.36	5.30 ± 1.01	9.35 ± 3.02	1.33 ± 0.06	1.00 ± 0.00	0.33 ± 0.03
Model	4.36 ± 0.35 ^∆∆^	5.27 ± 0.50 ^∆∆^	7.53 ± 1.51^∆^	14.44 ± 3.74	1.90 ± 0.27 ^∆∆^	5.13 ± 1.93 ^∆∆^	0.42 ± 0.05 ^∆∆^

^∆^ *p* < 0.05, ^∆∆^ *p* < 0.01, compared with normal group.

**Table 2:** The regression equation, linear range, and LLOQ of the seven components in rat plasma samples.

Components	Regression Equation	*R*^2^	Linear Range (ng mL^−1^)	LLOQ (ng mL^−1^)
hydroxysafflor yellow A	Y = 1.96 × 10^−4^X + 2.43 × 10^−4^	0.9959	1.125–225	1.125
caffeic acid	Y = 1.71 × 10^−3^X − 3.77 × 10^−3^	0.9988	1.145–229	1.145
*p*-coumaric acid	Y = 2.19 × 10^−3^X + 3.90 × 10^−4^	0.9987	1.065–1065	1.065
kaempferol-3-*O*-rutinoside	Y = 1.20 × 10^−3^X + 5.62 × 10^−3^	0.9989	1.37–685	1.37
ferulic acid	Y = 1.00 × 10^−3^X + 9.83 × 10^−4^	0.9975	4.5–900	4.5
3-*n*-butylphthalide	Y = 3.08 × 10^−2^X − 1.41 × 10^−1^	0.9986	1.23–246	1.23
ligustilide	Y = 4.45 × 10^−3^X − 1.20 × 10^−3^	0.9943	0.965–386	0.965

**Table 3:** Precision, accuracy, extraction recovery, matrix effect, and stability of the seven components in rat plasma samples (*n* = 6).

Components	Concentration (ng mL^−1^)	Inter-Day		Intra-Day		Extraction Recovery (%, Mean ± SD)	Matrix Effect (%, Mean ± SD)	Stability (RE/RSD%)		
		Precision (RSD%)	Accuracy (RE%)	Precision (RSD%)	Accuracy (RE%)			Short-Term	Three Freeze-Thaw Cycles	Long-Term
**1**	180	1.15	0.13	1.52	−0.40	86.83 ± 3.04	88.99 ± 1.86	0.55/1.25	−0.01/1.26	1.12/1.40
	22.5	1.72	0.58	2.39	−0.16	93.79 ± 3.79	87.06 ± 1.91	0.50/1.09	1.09/1.25	0.59/2.04
	2.25	4.04	−1.31	3.52	1.52	88.13 ± 3.10	88.99 ± 3.82	2.15/5.19	−1.01/3.96	0.41/5.29
**2**	183.2	1.88	0.77	1.24	0.23	91.42 ± 5.86	88.44 ± 2.98	1.12/1.93	0.27/2.28	1.28/1.72
	22.9	2.56	−0.82	3.38	−1.01	93.42 ± 5.63	86.91 ± 0.91	−2.01/3.12	−2.78/2.73	−3.21/2.92
	2.29	5.42	−4.27	4.22	−1.18	88.56 ± 1.70	87.56 ± 1.44	−6.50/8.48	−6.97/8.76	−5.68/7.73
**3**	852	1.84	1.69	1.74	2.11	87.76 ± 2.10	86.18 ± 2.11	1.64/1.62	1.87/2.27	1.78/1.94
	106.5	0.73	−2.97	3.62	−1.02	88.56 ± 0.91	89.83 ± 4.58	−0.53/3.65	−0.09/4.43	1.04/2.04
	2.13	3.67	1.02	3.60	0.37	95.72 ± 2.47	87.29 ± 1.99	−2.27/7.63	−2.22/8.02	−6.33/8.79
**4**	548	1.83	−0.03	2.49	0.85	87.72 ± 2.63	85.97 ± 1.51	2.18/2.14	1.26/2.61	1.51/2.76
	68.5	4.70	2.53	2.63	3.05	87.51 ± 1.66	87.24 ± 1.95	0.28/4.30	1.95/3.17	3.60/3.68
	2.74	4.19	−1.00	6.34	−3.22	89.91 ± 2.84	85.08 ± 7.66	−0.11/6.65	−3.67/9.31	−6.24/7.05
**5**	720	1.40	0.49	2.18	−0.53	86.68 ± 1.62	86.92 ± 3.29	1.27/2.22	−1.20/1.99	0.73/2.14
	90	1.47	2.33	3.62	−0.16	86.39 ± 5.10	88.07 ± 3.18	0.11/2.56	2.76/1.28	0.74/3.72
	9	2.47	2.10	4.86	0.45	87.03 ± 3.94	86.02 ± 1.96	14.13/7.65	−9.21/7.66	−0.43/7.72
**6**	196.8	1.85	0.69	1.04	1.52	88.68 ± 2.00	89.15 ± 4.60	1.23/1.64	2.18/1.13	0.89/2.03
	24.6	2.04	0.64	2.90	−0.09	88.42 ± 0.82	85.77 ± 2.99	0.48/3.14	−2.12/3.72	0.60/3.10
	2.46	3.40	−0.75	5.42	−0.13	87.45 ± 1.55	87.67 ± 3.36	2.41/6.57	−8.06/7.65	0.96/9.26
**7**	308.8	2.14	0.67	2.59	0.62	90.20 ± 0.86	86.28 ± 2.03	0.04/1.00	0.59/1.67	0.39/1.23
	38.6	2.08	2.15	2.56	0.84	88.30 ± 1.09	86.73 ± 4.23	1.60/1.90	1.84/2.46	1.33/2.07
	1.93	6.50	1.25	5.26	−1.25	87.61 ± 0.62	89.80 ± 8.21	3.17/9.21	7.58/8.45	4.13/6.92

**Table 4:** NCA pharmacokinetic parameters of seven components of Gui-Hong extracts between normal and blood stasis rats (*n* = 6).

Components	Group	*C*_max_ (ng mL^−1^)	*T*_max_ (h)	*T*_1/2z_ (h)	*MRT*_0-t_ (h)	*AUC*_0-t_ (ng mL^−1^ h)	*AUC*_0-∞_(ng mL^−1^ h)
**1**	normal	24.937 ± 8.883	1.833 ± 0.408	2.591 ± 0.3	3.653 ± 0.272	116.415 ± 31.299	126.972 ± 31.524
	model	43.62 ± 19.132	1.167 ± 0.408 *	4.075 ± 0.855 **	3.846 ± 0.27	187.687 ± 45.794 *	234.492 ± 69.678 **
**2**	normal	20.165 ± 5.345	0.75 ± 0.000	2.7 ± 1.161	2.212 ± 0.206	41.309 ± 3.209	53.374 ± 10.783
	model	63.905 ± 16.832 **	0.75 ± 0.000	2.721 ± 0.703	2.89 ± 0.267 **	165.157 ± 77.583 **	177.222 ± 76.372 **
**3**	normal	749.709 ± 28.526	0.167 ± 0.000	1.038 ± 0.374	0.759 ± 0.109	430.468 ± 33.622	434.338 ± 35.425
	model	811.579 ± 34.445 **	0.167 ± 0.000	1.104 ± 0.447	1.079 ± 0.135 **	706.649 ± 45.829 **	720.461 ± 53.934 **
**4**	normal	17.277 ± 2.931	0.75 ± 0.000	0.995 ± 0.527	1.727 ± 0.214	31.132 ± 7.886	32.162 ± 7.77
	model	19.686 ± 3.955	0.75 ± 0.000	2.253 ± 0.254 **	2.197 ± 0.104 **	41.06 ± 5.99 *	49.353 ± 5.514 **
**5**	normal	340.535 ± 34.027	0.167 ± 0.000	2.379 ± 1.096	1.431 ± 0.191	272.75 ± 65.418	311.633 ± 77.29
	model	392.586± 38.43 *	0.167 ± 0.000	1.85 ± 0.275	1.417 ± 0.236	297.676 ± 27.747	319.448 ± 31.097
**6**	normal	21.227 ± 10.74	0.167 ± 0.000	7.604 ±1.731	2.547 ±0.079	48.091 ± 8.745	108.678 ± 24.651
	model	25.127 ± 9.177	0.167 ± 0.000	5.338 ± 1.724 *	2.47 ± 0.166	62.826 ± 11.975 *	112.425 ± 27.906
**7**	normal	136.395 ± 25.05	0.083 ± 0.000	6.325 ± 1.453	5.693 ± 0.758	432.488 ± 71.117	463.314 ± 79.027
	model	162.474 ± 23.267	0.083 ± 0.000	5.772 ± 1.862	5.57 ± 0.262	461.85 ± 72.942	489.422 ± 92.129

* p < 0.05, ** *p* < 0.01, compared with the normal group.

**Table 5:** The retention time (t_R_), optimized MS/MS transitions, cone voltage, and collision energy for each component.

Components	Retention Time (min)	ESI Mode	MRM Transitions (Precursor-Product)	Cone Voltage (V)	Collision Energy (eV)
**1**	3.27	−	611.223→325.056	32	28
**2**	3.49	+	181.03→88.89	12	26
**3**	3.79	−	162.968→119.025	20	14
**4**	3.92	−	593.223→284.818	40	28
**5**	3.94	−	193.032→133.995	22	14
**6**	6.42	+	191.16→145.091	14	14
**7**	6.68	+	191.16→173.117	22	16
**8**	3.78	−	320.845→151.922	20	20
**9**	4.86	+	748.585→158.113	28	26
